# Identification of circulating T‐cell immunoglobulin and mucin domain 4 as a potential biomarker for coronary heart disease

**DOI:** 10.1002/mco2.320

**Published:** 2023-07-08

**Authors:** Mengyao Wang, Ke Gong, Xinran Zhu, Shasha Chen, Jie Zhou, Hui Zhang, Jihong Han, Likun Ma, Yajun Duan

**Affiliations:** ^1^ Key Laboratory of Metabolism and Regulation for Major Diseases of Anhui Higher Education Institutes, College of Food and Biological Engineering Hefei University of Technology Hefei China; ^2^ Department of Cardiology The First Affiliated Hospital of USTC Division of Life Sciences and Medicine University of Science and Technology of China Hefei China; ^3^ Key Laboratory of Bioactive Materials of Ministry of Education College of Life Sciences State Key Laboratory of Medicinal Chemical Biology Nankai University Tianjin China

**Keywords:** ADAM17, coronary heart disease, inflammation, p38, TIMD4

## Abstract

Efferocytosis, the process of engulfing and removing apoptotic cells, is attenuated in vulnerable plaques of advanced atherosclerosis. T‐cell immunoglobulin and mucin domain 4 (TIMD4) is a recognition receptor protein for efferocytosis that has been implicated in atherosclerosis mouse models. However, the role of serum‐soluble TIMD4 (sTIMD4) in coronary heart disease (CHD) remains unknown. In this study, we analyzed serum samples collected from two groups: Group 1 (36 healthy controls and 70 CHD patients) and Group 2 (44 chronic coronary syndrome [CCS]) and 81 acute coronary syndrome [ACS] patients). We found that sTIMD4 levels in patients with CHD were significantly higher than those in healthy controls and were also higher in ACS than in CCS patients. The area under the receiver operating characteristic curve was 0.787. Furthermore, our in vitro results showed that low‐density lipoprotein/lipopolysaccharide activated p38 mitogen‐activated protein kinase, which in turn enhanced a disintegrin and metalloproteinase 17, resulting in increased secretion of sTIMD4. This impairment of macrophage efferocytosis promoted inflammation. Thus, this study is not only the first identification of a potential novel biomarker of CHD, sTIMD4, but also demonstrated its pathogenesis mechanism, providing a new direction for the diagnosis and treatment of CHD.

## Graphical abstract

Efferocytosis is inextricably linked to atherosclerosis. TIMD4, a membrane protein mediating efferocytosis, can be cleaved into sTIMD4 by ADAM17. Here, we first reported that sTIMD4 in serum may serve as a biomarker for coronary heart disease. In vitro experiments demonstrated that sTIMD4 formation may be facilitated by activation of p38 mitogen‐activated protein kinase to activate ADAM17, thereby increasing the shearing of TIMD4 in an inflammatory environment.

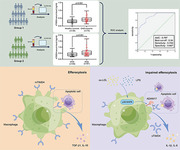

## 1 INTRODUCTION

Cardiovascular disease and its complications remain a leading cause of mortality and morbidity worldwide.^1^ Atherosclerosis, an inflammatory disease caused by lipid disorders, is determined by the balance between pro‐ and anti‐inflammatory processes.^2^ Efferocytosis, the clearance of apoptotic cells by macrophages, plays a critical role in the development of atherosclerosis by decreasing foam cells aggregation, inhibiting the production of reactive oxygen species and proinflammatory mediator production, and promoting anti‐inflammatory and antioxidant responses. Efferocytosis also induces the expression of anti‐inflammatory genes, such as interleukin‐10 (IL‐10), transforming growth factor‐β (TGF‐β), and macrophage polarization from type 1 to type 2.^3^

While phagocytosis and clearance of apoptotic cells are efficiency in the early stage of atherosclerosis, in the advanced stage, the inability of efferocytosis to clear apoptotic cells leads to the formation of a necrotic core, promoting the production of inflammatory factors and exacerbating the progression of atherosclerosis. The inadequate clearance of apoptotic cells by phagocytes may be an important determinant of clinical events in acute atherosclerotic thrombosis.^4^

Macrophage phagocytosis involves three steps: "find‐me," "eat‐me," and "engulfment and processing."^3^ The T cell immunoglobulin and mucin domain protein (TIMD) family comprises type 1 transmembrane proteins, with TIMD4 expressed predominantly in macrophages and dendritic cells.^5^ TIMD4 acts as a ligand of TIMD1 and recognizes phosphatidylserine on the surface of apoptotic cells, transmitting "eat‐me" signals and promoting macrophages to phagocytose apoptotic cells.^6^, ^7^ Foks et al. demonstrated that the blockade of TIMD4 increased atherosclerosis in high‐fat diet‐induced low‐density lipoprotein receptor knockout mice, possibly by inhibiting TIMD4 phagocytosis.^8^

Metalloproteinases mediate the posttranslational release of extracellular domains of diverse transmembrane proteins, including cytokines, growth factors, receptors, and adhesion molecules.^9^ A disintegrin and metalloproteinase 17 (ADAM17), a type I transmembrane metalloproteinase, mediates the shedding of several transmembrane proteins in the extracellular domain and is associated with various diseases, such as atherosclerosis, adipose tissue metabolism, insulin resistance and diabetes.^10^ Schweigert et al. identified TIMD4 as the substrate of ADAM17, which cleaves it into soluble TIMD4 (sTIMD4) molecules, although sTIMD4 can still bind phospholipid amide acid.^11^ STIMD4 can be determined in serum and body fluids, and its levels may indicate pathological status. For instance, in patients with ischemic stroke, plasma sTIMD4 levels increased on day 2 and 5, and the National Institute of Health stroke scale score was positively correlated with plasma sTIMD4 levels, suggesting that sTIMD4 may be a plasma prognostic biomarker for ischemic stroke.^12^ Similarly, plasma sTIMD4 levels in patients with ankylosing spondylitis were significantly higher than those in the control group, and positively correlated with plasma tumor necrosis factor‐α (TNF‐α) levels and Barth disease activity index of ankylosing spondylitis.^13^ However, the role of sTIMD4 in CHD is not well understood.

Therefore, the purpose of this study is to investigate the relationship between serum sTIMD4 levels and coronary heart disease (CHD) in order to elucidate their mechanism. We hypothesized that several factors, such as high oxidized low‐density lipoprotein (ox‐LDL), may activate ADAM17 to shear membrane TIMD4 (mTIMD4) protein on the surface, resulting in a decrease of mTIMD4 and an increase of sTIMD4 in serum, and thus reduces the clearance ability of macrophages and exacerbates the progression of atherosclerosis. Finally, elevated serum sTIMD4 may be a biomarker of CHD.

## 2 RESULTS

### 2.1 Serum sTIMD4 level was positively correlated with CHD events

The study recruited 106 participants, comprising 36 healthy controls and 70 CHD patients with exclusion of diabetes and nephropathy were recruited for blood sample collection and sTIMD4 level determination. Individual information was also collected for evaluation. Demographic and clinical characteristics of the study population were summarized in Table 1.

**TABLE 1:** Demographic and clinical data for CHD patients and healthy controls (Group 1).

Variable	Total (*N* = 106)	Healthy controls (*N* = 36)	CHD patients (*N* = 70)	*p* Value
Sex, male	**65 (61%)**	**16 (44%)**	**49 (70%)**	**0.041**
Average age (years)	**61 (49–68)**	**41 (33–60)**	**64 (56–72)**	**<0.001**
Bodyweight (kg)	**66.0 (57.0–76.3)**	**63.3 (55.4–69.68)**	**69 (59.0–79.0)**	**0.013**
BMI (kg/m^2^)	**24.25 ± 3.70**	**22.43 ± 3.23**	**25.19 ± 3.58**	**<0.001**
Blood glucose (mmol/L)	5.03 (4.57–5.45)	5.09 (4.83–5.43)	4.92 (4.44–5.47)	0.190
LDL‐C (mmol/L)	2.34 ± 0.66	2.46 ± 0.39	2.27 ± 0.76	0.167
HDL‐C (mmol/L)	**1.16 (0.93–1.40)**	**1.40 (1.20–1.60)**	**0.97 (0.88–1.23)**	**<0.001**
T‐CHO (mmol/L)	4.20 ± 0.89	4.26 ± 0.65	4.17 ± 0.99	0.605
TG (mmol/L)	**1.28 (0.95–1.84)**	**1.11 (0.78–1.34)**	**1.37 (1.06–2.06)**	**0.002**
AI	**2.80 (1.99–3.28)**	**2.08 (1.69–2.79)**	**2.99 (2.47–3.70)**	**<0.001**
Systolic BP (mmHg)	**128 ± 20**	**122 ± 21**	**132 ± 19**	**0.011**
Diastolic BP (mmHg)	79 (70–88)	78 (66–87)	81 (72–90)	0.122
Diabetes	0 (0%)	0 (0%)	0 (0%)	N/A
sTIMD4 (ng/mL)	**0.36 (0.26–0.47)**	**0.28 (0.21–0.39)**	**0.42 (0.31–0.53)**	**<0.001**

Abbreviations: AI, atherosclerosis index; BMI, body mass index; BP, blood pressure; HDL‐C, high‐density lipoprotein cholesterol; LDL‐C, low‐density lipoprotein cholesterol; T‐CHO, total cholesterol; TG, total triglyceride. *p* < 0.05 was considered significant.

**FIGURE 1:**
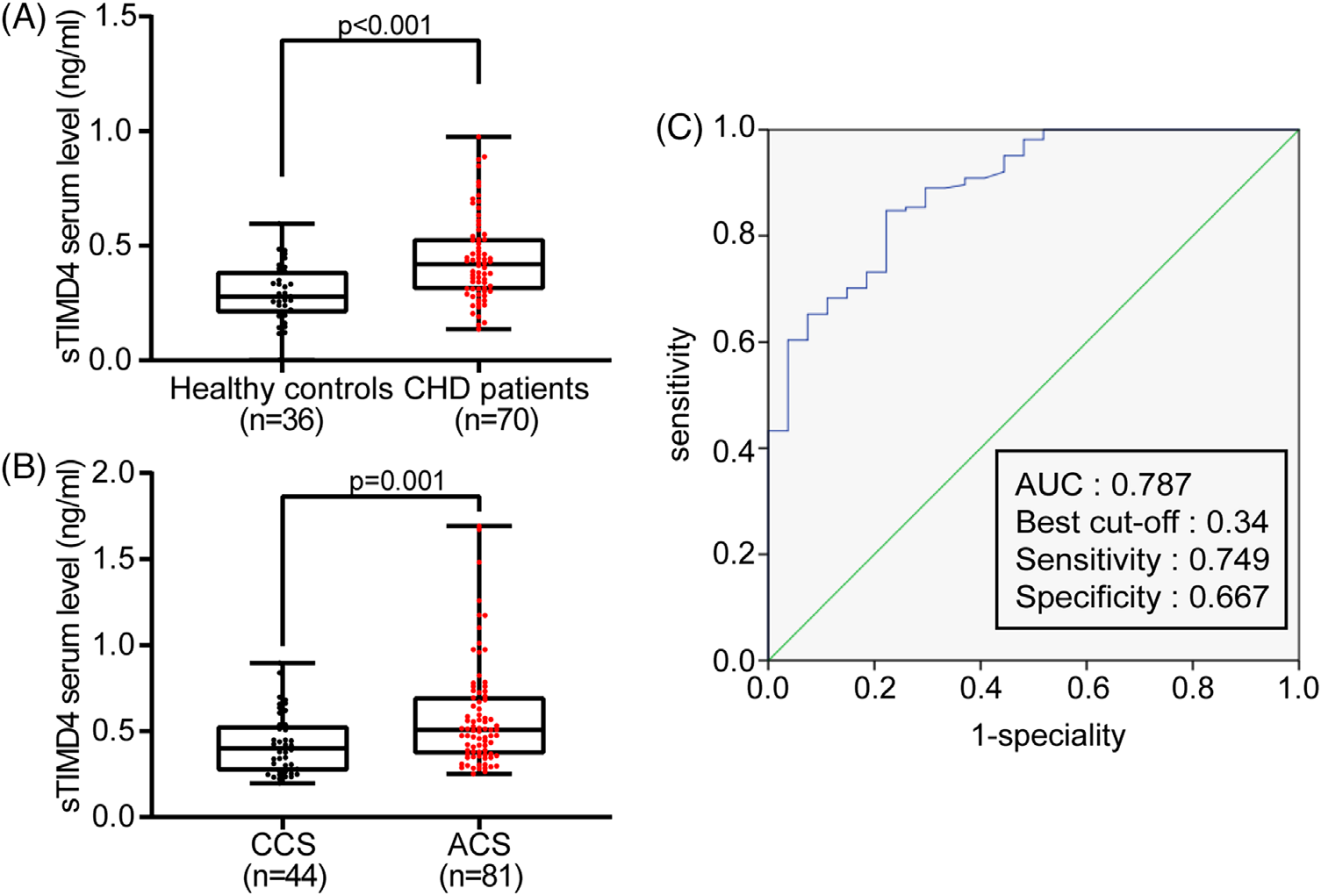
Serum sTIMD4 levels were increased associated with CHD. Serum samples collected from healthy controls, CHD, CCS, or ACS patients were used to determine TIMD4 levels by ELISA, followed by comparison with nonparametric tests between healthy controls and CHD patients (Group 1, A) or CCS and ACS patients (Group 2, B). Each dot presents an individual. The whisker plot presents the mean with min to max, and the beard plot presents an average from smallest to largest. (C) ROC curve of serum sTIMD4 for predicting CHD events. AUC is 0.787. The optimal cut‐off is 0.34. The sensitivity is 0.749, and the specificity is 0.667.

Serum sTIMD4 levels were significantly higher in CHD patients than in healthy subjects (0.28 ng/mL [0.21−0.39] vs. 0.42 ng/mL [0.31−0.53], *p* < 0.001; Figure 1A). To rule out other factors, logistic regression analysis was conducted to determine whether serum sTIMD4 was independently and significantly associated with CHD events. Model 3 demonstrated that serum sTIMD4 was still an independent factor associated with CHD events (odds ratio: 1.008, 95% confidence interval [CI]: 1.001‐1.015, *p* = 0.021) after adjusting for age, sex, weight, body mass index (BMI), low‐density lipoprotein (LDL‐C), high‐density lipoprotein cholesterol (HDL‐C), total cholesterol (T‐CHO), triglycerides (TG), atherogenic index (AI), blood glucose, systolic and diastolic blood pressure (BP) (Table S2).

**FIGURE 2:**
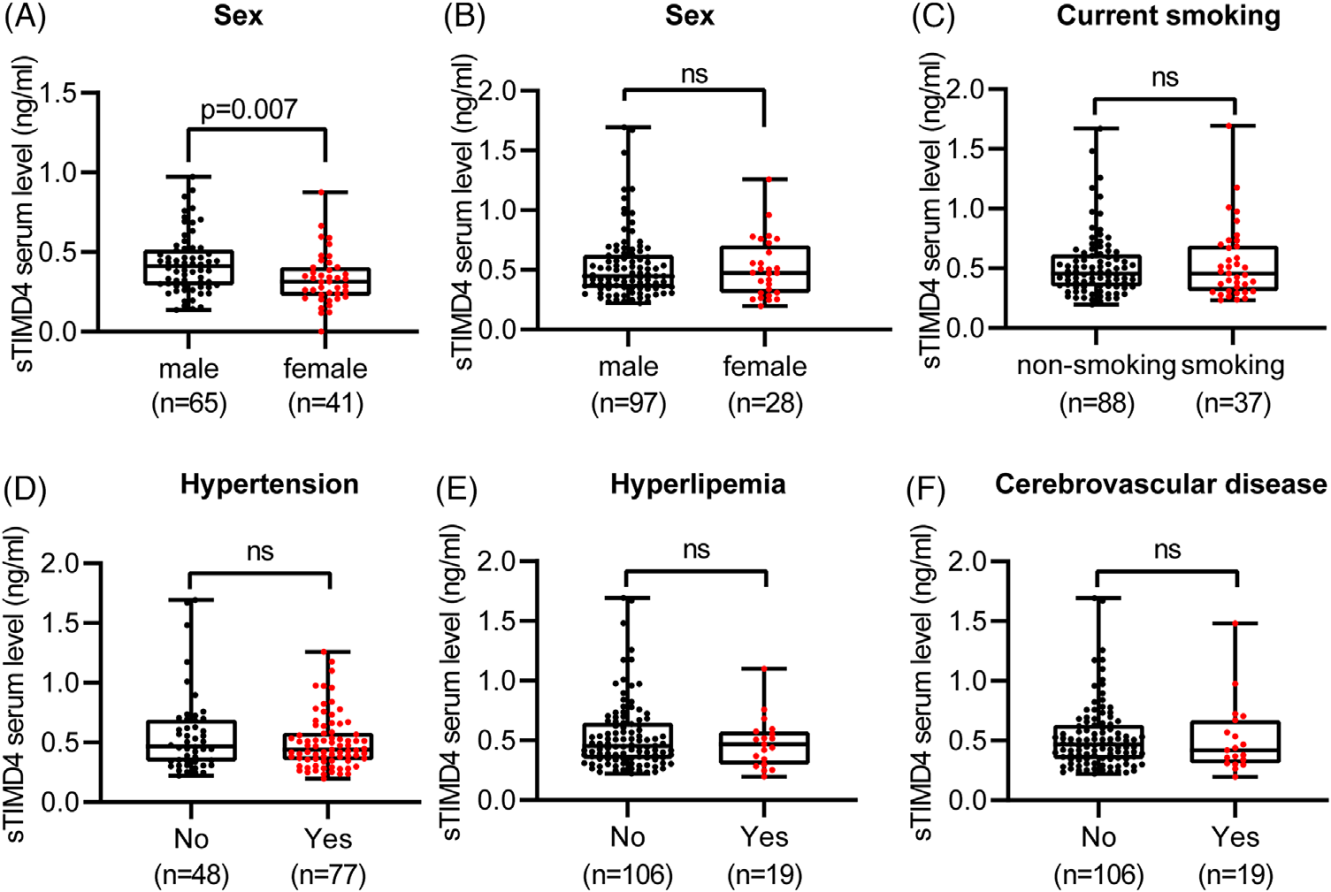
Sex, smoking, hypertension, hyperlipemia, and cerebrovascular diseases had no effect on serum sTIMD4 levels between CCS and ACS patients. Serum sTIMD4 levels in Figure 2 were used to compare between males and females in Group 1 and Group 2 (A and B), nonsmoking and smoking patients (C), patients with normotension and hypertension (D), patients with or without hyperlipemia (E), and patients with or without cerebrovascular disease (F) in Group 2. The individuals are represented by black and red dots, and the beard plot represents an average from smallest to largest. Nonparametric tests were used for comparison between group of CCS and ACS. ns, not significant.

In addition, Spearman's rank correlation coefficient was used to detect the correlation between serum sTIMD4 levels and cardiometabolic risk factors. Table S4 showed that serum sTIMD4 levels were correlated with age (*p* = 0.023), bodyweight (*p* < 0.001), BMI (*p* < 0.001), AI (*p* < 0.001), HDL‐C (*p* < 0.001), and TG (*p* < 0.001). Moreover, serum sTIMD4 levels were significantly higher in males than in females (*p* = 0.007; Figure 2A). Taken together, these findings suggest that serum sTIMD4 may be a risk factor for CHD.

### 2.2 Serum sTIMD4 level was associated with ACS events

The association between serum sTIMD4 level and acute coronary syndrome (ACS) was investigated in this study. ACS encompasses a group of acute cardiac ischemia syndromes caused by thrombosis formed by rupture or erosion of unstable atherosclerotic plaques in the coronary, involving acute T‐segment elevation myocardial infarction (STEMI), acute non‐ST‐segment elevation myocardial infarction (NSTEMI), and unstable angina (UA).^14^ The reduced phagocytic ability of macrophages due to the involvement of TIMD4 in efferocytosis may lead to the accumulation of inflammatory necrotic cells in advanced atherosclerosis, reducing the stability of plaque, leading to plaque rupture and some acute cardiovascular events.

To evaluate the association between serum sTIMD4 and ACS, we recruited additional patients with CHD and excluded individuals with diabetes and nephropathy, including 44 diagnosed with chronic coronary syndrome (CCS) and 81 diagnosed with ACS. The demographic and clinical characteristics of the study population were presented in Table 2. We found that serum sTIMD4 levels were significantly higher in patients with ACS than in those with CCS (0.40 ng/mL [0.27−0.53] vs. 0.51 ng/mL [0.37−0.70], *p* = 0.001; Figure 1B). Logistic regression analysis was conducted to exclude other factors and determine whether serum sTIMD4 was associated with ACS event independently. After adjusting for potential confounders, serum sTIMD4 was still an independent risk factor for ACS event (odds ratio: 1.502, 95% CI: 1.139–1.981, *p* = 0.004) (Table S3).

**TABLE 2:** Demographic and clinical data for CCS and ACS patients (Group 2).

Variable	Total (*N* = 125)	CCS (*N* = 44)	ACS (*N* = 81)	*p* Value
Sex, male	97 (78%)	36 (82%)	61 (75%)	0.404
Age (years)	62 ± 9	61 ± 8	62 ± 9	0.353
Bodyweight (kg)	69 ± 10	71 ± 11	68 ± 10	0.188
BMI (kg/m^2^)	25.0 ± 2.6	25.5 ± 2.9	24.7 ± 2.4	0.117
Blood glucose (mmol/L)	4.93 (4.43–5.35)	4.92 (4.46–5.25)	4.98 (4.37–5.45)	0.288
LDL‐C (mmol/L)	2.02 (1.56–2.79)	1.97 (1.70–2.79)	2.08 (1.54–2.81)	0.591
HDL‐C (mmol/L)	1.03 (0.91–1.16)	1.06 (0.94–1.21)	1.02 (0.89–1.14)	0.328
T‐CHO (mmol/l)	3.94 (3.34–4.93)	3.97 (3.55–4.85)	3.91 (3.31–4.99)	0.573
TG (mmol/L)	1.34 (1.03–2.01)	1.24 (1.03–1.85)	1.43 (1.03–2.03)	0.482
AI	2.82 (2.18–4.03)	2.85 (2.07–4.01)	2.82 (2.18–4.03)	0.961
Systolic BP (mmHg)	136 ± 18	133 ± 19	137 ± 17	0.339
Diastolic BP (mmHg)	83 ± 10	83 ± 11	84 ± 10	0.646
Smoking	37 (30%)	14 (32%)	23 (28%)	0.689
Diabetes	0 (0%)	0 (0%)	0 (0%)	N/A
Hypertension	77 (62%)	27 (61%)	50 (62%)	0.968
Cerebrovascular disease	19 (15%)	7 (19%)	12 (15%)	0.871
Hyperlipemia	19 (18%)	4 (9%)	15 (19%)	0.161
sTIMD4 (ng/mL)	**0.46 (0.35–0.63)**	**0.40 (0.27–0.53)**	**0.51 (0.37–0.70)**	**0.001**

Abbreviations: AI, atherosclerosis index; BMI, body mass index; BP, blood pressure; HDL‐C, high‐density lipoprotein‐cholesterol; LDL‐C, low‐density lipoprotein‐cholesterol; TC, total triglyceride; T‐CHO, total cholesterol. *p* < 0.05 was considered significant.

Spearman correlation coefficient was used to analyze the association between serum sTIMD4 level and other indicators, which did not show any significant correlation (Table S4). Furthermore, there was also no significant difference in serum sTIMD4 levels between men and women, regardless of whether they smoked, had hypertension, hyperlipidemia, or had cerebrovascular disease (Figures 2B–F). These findings suggested that serum sTIMD4 may serve as an independent risk factor for ACS events in patients with CHD.

### 2.3 Diagnostic value for serum sTIMD4 in distinguishing healthy controls from CHD patients

The diagnostic value of serum sTIMD4 in distinguishing healthy controls from CHD patients was evaluated by conducting receiver operating characteristic (ROC) curve analysis. The area under the curve (AUC) was found to be 0.787 (Figure 1C). The optimal cut‐off value for sTIMD4 was 0.34 ng/mL, at which the sum of sensitivity and specificity was maximum in discriminating healthy controls from CHD patients. The sensitivity and specificity were 74.9 and 66.7%, respectively, with an overall accuracy of 74%. Positive predictive value for sTIMD4 at 0.34 ng/mL was 93%, while the negative predictive value was only 33.8%, possibly due to the limited sample size of healthy controls (Table S5). In summary, serum sTIMD4 was found to be a valuable biomarker for distinguishing healthy controls from CHD patients.

### 2.4 Ox‐LDL increased ADAM17 and sTIMD4 while decreased mTIMD4

**FIGURE 3:**
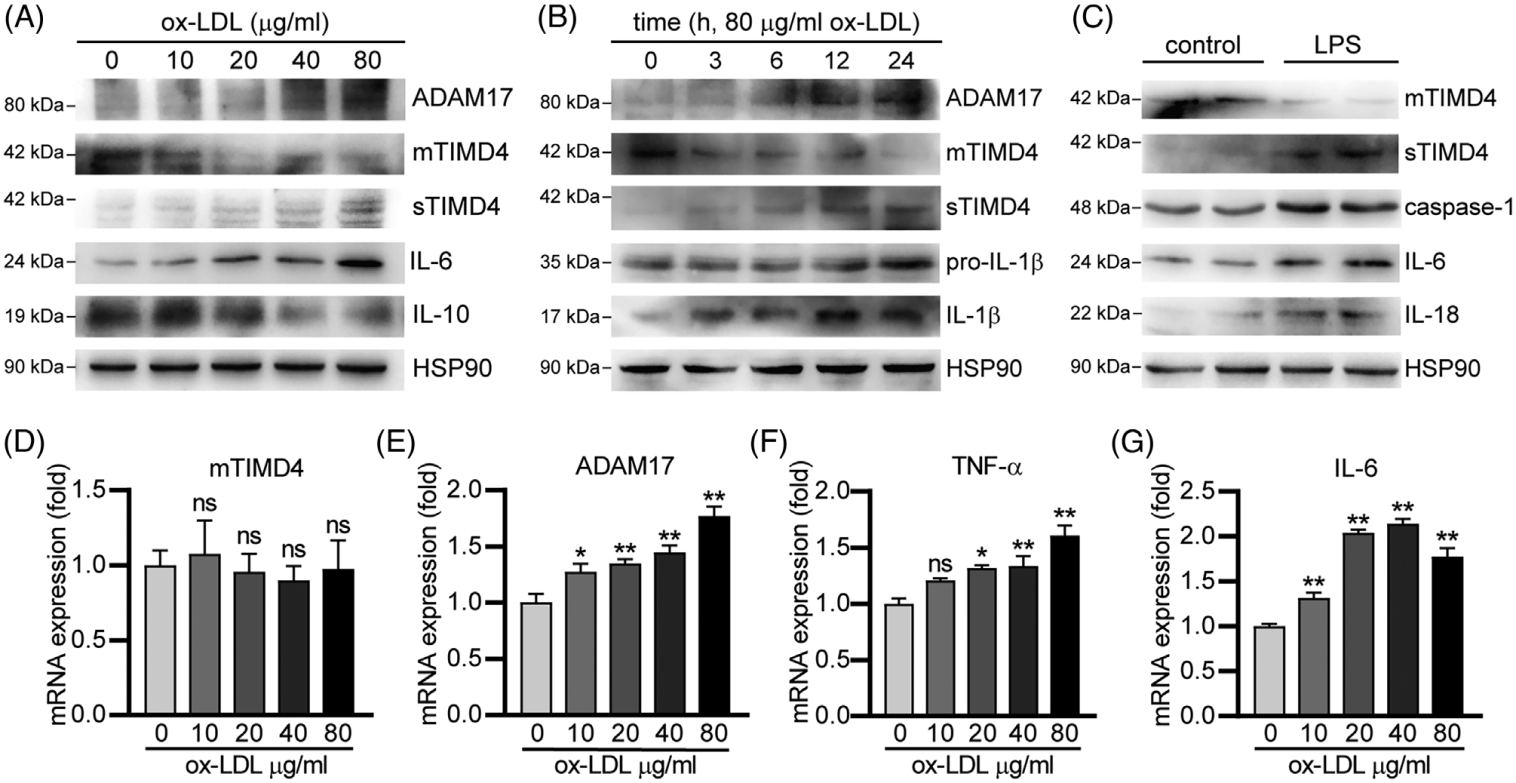
Ox‐LDL increased ADAM17 and sTIMD4 while decreased mTIMD4 level in macrophages. RAW264.7 cells were treated with ox‐LDL at the indicated concentrations for 24 h (A, D–G) or with 80 μg/mL ox‐LDL for the indicated times (B) or LPS at 1 μg/mL for 24 h (C). After treatment, total cellular proteins, RNA, and treatment mediums were collected. (A–C) Expression of ADAM17, mTIMD4, IL‐6, IL‐10, pro‐IL‐1β, IL‐1β, caspase‐1, and IL‐18 in cells and sTIMD4 in medium was detected by Western blotting (*n* = 3). (D and E) Expression of TIMD4, ADAM17, TNF‐α, and IL‐6 mRNA was determined by qRT‐PCR (*n* = 3–4). HSP90 was used as a loading control. *p < 0.05; **p < 0.01; ns, not significant.

We investigated whether the increase of sTIMD4 in serum of patients with CHD, is related to the shearing of ADAM17, which cleaves the extracellular domain structure of human TIMD4 protein and generates a soluble form of TIMD4.^11^ To do so, we treated RAW264.7 cells with ox‐LDL, which is an in vitro atherosclerotic cell model, for different times (0, 3, 6, 12, 24 h) or at different concentrations (0, 10, 20, 40, 80 μg/mL). Results showed that the levels of mTIMD4 decreased, while the levels of ADAM17 and sTIMD4 increased by in a gradient manner in response to ox‐LDL, accompanied by high expression of proinflammatory factors, such as IL‐6 and IL‐1β, and low expression of anti‐inflammatory factors, such as IL‐10 (Figures 3A and B). Furthermore, ox‐LDL increased the mRNA expression of ADAM17, IL‐6, and TNF‐α, while it did not affect mTIMD4 mRNA expression (Figures 3D–G), suggesting that ox‐LDL may regulate TIMD4 at the protein level.

As a known immune checkpoint protein, TIMD4 plays an important role in the immune response of atherosclerosis.^15^ Thus, we stimulated RAW264.7 cells with lipopolysaccharide (LPS) to explore the changes of TIMD4 in an inflammatory environment. LPS stimulates caspase‐1 to cleave the full‐length precursor prointerleukin‐18 (pro‐IL‐18), thereby hydrolyzing them into biologically active fragments, which is an important process in inflammatory responses.^16^ Hence, we determined that the expression of IL‐18 and caspase‐1 as well as IL‐6 was significantly increased by LPS associated with enhanced sTIMD4 level and reduced mTIMD4 level (Figure 3C). These results suggest that mTIMD4 cleavage may also be related to the production of inflammatory factors.

### 2.5 Ox‐LDL or LPS activated ADAM17 to shear mTIMD4

**FIGURE 4:**
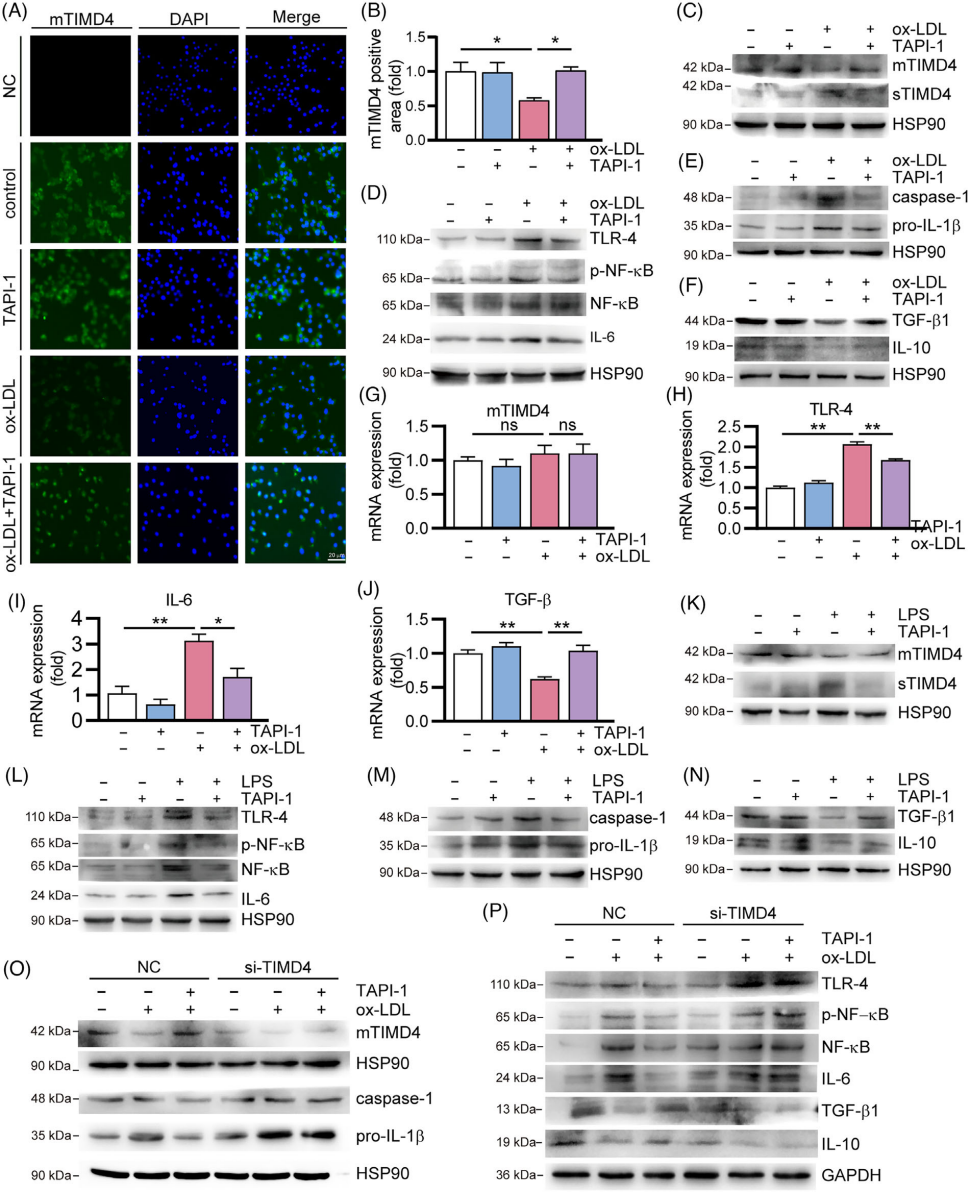
Ox‐LDL/LPS activated ADAM17 to increase cleavage of mTIMD4 in macrophages. RAW264.7 cells treated with ox‐LDL (80 μg/mL) and TAPI‐1 (1 μM) for 24 h. (A) and (B) mTIMD4 expression was detected by immunofluorescent staining (*n* = 5). (C–F) The protein expression of mTIMD4, sTIMD4 in medium, TLR‐4, p‐NF‐κB, NF‐κB, IL‐6, caspase‐1, pro‐IL‐1β, TGF‐β1, and IL‐10 were detected by Western blotting (*n* = 3). (G–J) The mRNA expression mTIMD4, TLR‐4, IL‐6, and TGF‐β1 was determined by qRT‐PCR (*n* = 3–4). (K–N) RAW264.7 cells were treated with LPS (1 μg/mL) and TAPI‐1 (1 μM) for 24 h, and the protein expression of mTIMD4, sTIMD4 in medium, TLR‐4, p‐NF‐κB, NF‐κB, IL‐6, caspase‐1, pro‐IL‐1β, TGF‐β1, and IL‐10 were detected by Western blotting (*n* = 3). (O–P) RAW264.7 cells were transfected with 20 nM control si‐NC or si‐TIMD4 for 48 h and incubated with ox‐LDL (80 μg/mL) and TAPI‐1 (1 μM) for 24 h, followed by determination of mTIMD4, TLR‐4, p‐NF‐κB, NF‐κB, IL‐6, IL‐10, TGF‐β1, caspase‐1, pro‐IL‐1β protein expression by Western blotting (*n* = 3). HSP90 or GAPDH was used as a loading control. ns, not significant. *p < 0.05; **p < 0.01.

To investigate the influence of ADAM17 on TIMD4 in the presence of ox‐LDL (80 μg/mL) or LPS (1 μg/mL), we treated RAW264.7 cells with TAPI‐1 (1 μM), an ADAM17 inhibitor. In the absence of TAPI‐1, ox‐LDL decreased mTIMD4 protein expression and increased sTIMD4 protein level. However, with TAPI‐1 treatment, mTIMD4 protein expression increased whiles sTIMD4 level decreased noticeably (Figures 4A–C), indicating that the process of ox‐LDL activating ADAM17 to shear mTIMD4 is ADAM17‐dependent. Additionally, TAPI‐1 abolished the protein expression of phosphorylated nuclear factor kappa B (p‐NF‐κB), anti‐Toll‐like receptor 4 (TLR‐4) and IL‐6 that were upregulated by ox‐LDL (Figure 4D). Moreover, the protein expression of caspase‐1 and pro‐IL‐1β was increased by ox‐LDL and reduced by TAPI‐1 (Figure 4E), whereas the protein expression of anti‐inflammatory factors (IL‐10 and TGF‐β1) was upregulated by TAPI‐1 in response to ox‐LDL (Figure 4F). TAPI‐1 also reduced the mRNA expression of TLR‐4, IL‐6, and TGF‐β1 induced by ox‐LDL (Figures 4H–J), but TIMD4 mRNA expression remained unchanged (Figure 4G). Similar results were obtained in the presence of LPS (1 μg/mL) (Figures 4K–N). These findings suggest that the inflammation caused by ox‐LDL or LPS is related to ADAM17 signaling, and that ADAM17 affects TIMD4 at protein level.

ADAM17 has been reported to have at least 90 substrates, including IL‐6 receptor, TNF‐α, and epidermal growth factor receptor in addition to TIMD4.^17^ To eliminate the effect of other ADAM17 substrates on inflammation, RAW264.7 cells were transfected with si‐TIMD4 (20 nM) for 48 h, followed by treatment with ox‐LDL (80 μg/mL) and TAPI‐1 (1 μM). Si‐TIMD4 significantly decreased mTIMD4 expression (Figure 4O). As demonstrated in Figures 4O and P, TAPI‐1 reduced the expression of inflammation related genes (TLR‐4, p‐NF‐κB, IL‐6, caspase‐1, and pro‐IL‐1β) and increased the expression of IL‐10 and TGF‐β1. However, after si‐TIMD4 treatment, the expression of TLR‐4, p‐NF‐κB, IL‐6, caspase‐1, pro‐IL‐1β, TGF‐β1, and IL‐10 was not evidently changed by TAPI‐1, indicating that the anti‐inflammatory effects of ADAM17 inhibition by TAPI‐1 were at least partially accomplished through TIMD4.

### 2.6 Ox‐LDL/LPS activated ADAM17 to shear TIMD4 through the p38 mitogen‐activated protein kinase signaling pathway

**FIGURE 5:**
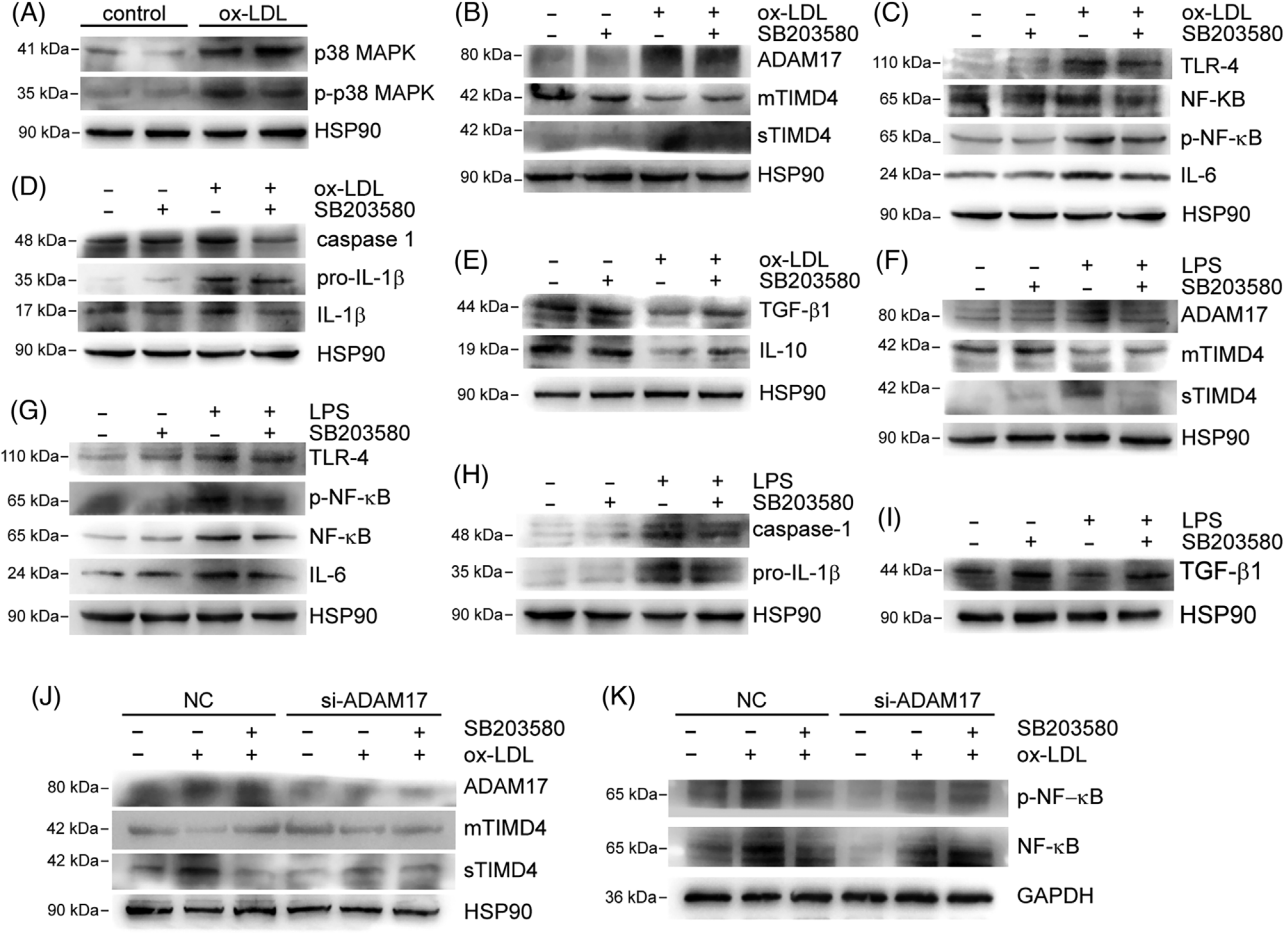
Ox‐LDL/LPS activated ADAM17 to shear mTIMD4 through the p38 MAPK signaling pathway. (A) RAW264.7 cells were treated with ox‐LDL (80 μg/mL), and the protein expression of p‐p38 MAPK and p38 MAPK were detected by Western blotting (*n* = 3). (B–E) RAW264.7 cells were treated with ox‐LDL (80 μg/mL) and SB203580 (10 μM) for 24 h, and the protein expression of mTIMD4, sTIMD4 in medium, ADAM17, TLR‐4, p‐NF‐κB, NF‐κB, IL‐6, caspase‐1, pro‐IL‐1β, IL‐1β, TGF‐β1, and IL‐10 were detected by Western blotting (*n* = 3). (F–I) RAW264.7 cells treated with LPS (1 μg/mL) and SB203580 (10 μM) for 24 h, and the protein expression of mTIMD4, sTIMD4 in medium, ADAM17, TLR‐4, NF‐κB, p‐NF‐κB, IL‐6, caspase‐1, pro‐IL‐1β, and TGF‐β1 was detected by Western blotting (*n* = 3). (J–K) RAW264.7 cells were transfected with 20 nM control si‐NC or si‐ADAM17 for 48 h, followed by determination of ADAM17, mTIMD4, sTIMD4, p‐NF‐κB, NF‐κB protein expression by Western blotting (*n* = 3). HSP90 or GAPDH was used as a loading control.

Our findings indicate that the activation of the p38 mitogen‐activated protein kinase (MAPK) signaling pathway by ox‐LDL/LPS leads to the cleavage of TIMD4 by ADAM17. Specifically, we observed an increase in the expression of p‐p38, p38, ADAM17, and sTIMD4, and a decrease in the expression of mTIMD4 in RAW264.7 cells treated with ox‐LDL for 24 h (Figures 5A and B).

To further investigate the role of the p38 MAPK signaling pathway in this process, we treated RAW264.7 cells with both ox‐LDL and SB203580, an inhibitor of p38 MAPK. We observed a decrease in the expression of ADAM17 and sTIMD4, and an increase in the expression of mTIMD4 (Figure 5B). Moreover, the inflammation induced by ox‐LDL was alleviated by SB203580, as shown in Figures 5C–E. Similarly, treating RAW264.7 cells with LPS and SB203580 also yielded consistent results consistent results (Figures 5F–I). In addition, we treated RAW264.7 cells with si‐ADAM17 and SB203580 to determine whether the role of p38 MAPK on TIMD4 is mediated by ADAM17. When ADAM17 was knocked down, SB203580 no longer significantly reduced the sTIMD4 level and increased the mTIMD4 level significantly in the presence of ox‐LDL. Similarly, the inhibitory effect of SB203580 on inflammation was also reduced (Figures 5J and K).

**FIGURE 6:**
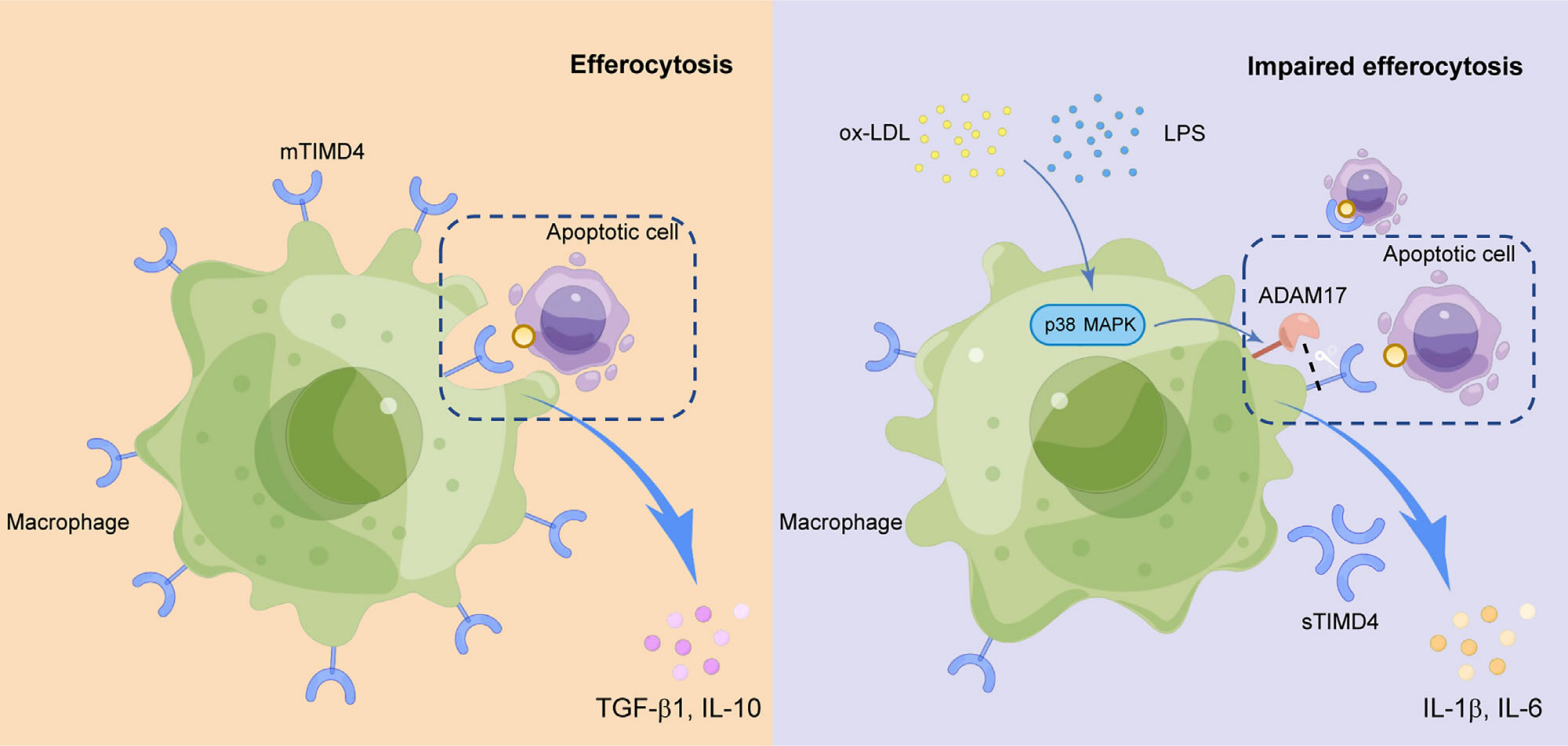
Mechanism schematic diagram of sTIMD4 formation. Under normal conditions, mTIMD4 on the surface of macrophage cell membranes recognizes phosphatidylserine on apoptotic cells for efferocytosis, which increases anti‐inflammatory cytokines TGF‐β1 and IL‐10 (left). When stimulated by ox‐LDL or LPS, p38 MAPK is activated in macrophages, thereby activating ADAM17 to shear mTIMD4, resulting in impaired efferocytosis and elevated sTIMD4 levels along with the increase in inflammatory mediators IL‐1β and IL‐6 (right). Figure 6 was plotted at https://www.figdraw.com (export ID: AAOSU7d762).

Taken together, our results suggest that in macrophages, ox‐LDL or LPS activates the p38 MAPK signaling pathway, which in turn promotes the expression of ADAM17 and sTIMD4 and the cleavage of mTIMD4 leading to inflammation (Figure 6).

## 3 DISCUSSION

In this study, we observed that the levels of sTIMD4 were elevated in the serum of CHD patients compared with healthy controls. Furthermore, we found that sTIMD4 levels were even higher in ACS patients compared with CCS patients. Our ROC analysis suggests that sTIMD4 could serve as a promising biomarker for the diagnosis of CHD.

TIMD4, a membrane protein receptor, has been implicated in various physiological processes, including lipid metabolism and immune system regulation.^18^ Previous studies have demonstrated that TIMD4‐mediated efferocytosis is closely associated with atherosclerosis.^19^ Efferocytosis plays a crucial role in maintaining tissue homeostasis by facilitating the clearance of apoptotic cells through specific phagocytic receptors.^20^ In the late stage of atherosclerosis, impaired efferocytosis of macrophages leads to the accumulation of apoptotic cells and foam cells, which contribute to the generation of necrotic lipid cores and increased plaque vulnerability.^21^ Dysfunctional surface receptors on apoptotic cells or phagocytes are often cleaved by enzymes and shed from the cell surface into the circulation.^22^, ^23^, ^24^, ^25^ Soluble forms of membrane proteins have emerged as potential biomarkers for several diseases, including atherosclerosis.^20^ Most previous studies have focused on the impact of TIMD4‐mediated efferocytosis effects on disease,^8^, ^26^, ^27^ without explaining why efferocytosis is impaired in certain disease. In our study, we found that ADAM17‐mediated cleavage of mTIMD4 by p38 MAPK activation leads to an increase in sTIMD4 levels and impaired efferocytosis in macrophages treated with ox‐LDL or LPS. These findings provide a mechanistic basis for the elevated sTIMD4 levels observed in clinical CHD patients.

The results of our study suggest that sTIMD4 may be a promising biomarker for diagnosing CHD, especially for identifying patients with ACS. While biomarkers of CHD have been extensively studied, the pathogenesis of the disease is complex and multifactorial.^28^ Thus, the discovery of new systemic biomarkers and therapeutic targets is necessary. Our study sheds light on the potential utility of a novel biomarker for CHD, which may aid in the development of more effective diagnostic and therapeutic strategies.

The deficiency of ADAM17 in macrophage has been shown to enhance CD36‐dependent efferocytosis and anti‐inflammatory effects,^29^ and ADAM17 inhibitors have been reported to reduce the release of soluble TNF‐α.^30^ Our results indicated that TAPI‐1, an ADAM17 inhibitor, significantly increased the level of mTIMD4 on cell membranes while reducing the level of sTIMD4 in supernatant, effectively inhibiting inflammatory response in vitro. However, when TIMD4 was knocked down, this anti‐inflammatory effect was significantly subdued, suggesting that ADAM17 may be upstream of TIMD4 in the inflammatory microenvironment. Previous research has demonstrated that ADAM17‐mediated extracellular domain cleavage is activated by p38 MAPK and extracellular signal‐regulated kinase signal.^31^ Hypoxia has been shown to activate p38 MAPK to enhance ADAM17 expression and activity, inducing keratinocyte migration,^32^ while angiotensin II activated the p38 MAPK/ADAM17 pathway to promote Mer tyrosine kinase cleavage, facilitating atherosclerosis.^33^ In a human model of acute skin inflammation, increased activity of macrophage p38 MAPK led to a decrease in TIMD4 in older adults than in younger ones. Active p38 MAPK inhibitors administered orally in the elderly salvaged TIMD4 expression, cleared apoptotic bodies and restored the decomposable phenotype of macrophages.^27^ Our results also confirmed that p38 MAPK regulated ADAM17‐mediated exo‐domain shedding in a cellular model of atherosclerosis.

It is noteworthy that the cleavage of membrane proteins occurs in many biological processes. For instance, the immune receptor killer cell lectin like receptor K1 (NKG2D) activates CD8^+^T cells and natural killer (NK) cells to stimulate tumor immunity by binding to corresponding ligands on tumor cells.^34^, ^35^ Major histocompatibility complex class I polypeptide‐related sequence A/B (MICA/B), its ligand, has been shown to be widely expressed in human tumors.^36^ Interestingly, MICA/B is also cleaved by metalloproteinases to form a soluble form,^37^ and the determination of soluble MICA levels can be used as an immunological diagnostic marker in patients with epithelial malignant tumors.^38^ Tumor cells escape NKG2D‐mediated tumor immune monitoring by promoting MICA/B shedding, which is often referred to as tumor immune escape.^39^, ^40^ Badrinath et al. prepared an antibody targeting the shear site of MICA/B to protect it from being cleaved, allowing NK cells and CD8^+^ T cells to play their normal roles and kill tumor cells.^41^ Interestingly, the function of MICA/B seems to be similar to that of TIMD4. Therefore, whether we can also prepare a TIMD4 antibody to protect it from being cleaved by ADAM17 and promote the efferocytosis of macrophages to alleviate atherosclerosis, which will be our future research direction.

However, there are several limitations to this study. First, although multiple proximity extension analysis has been widely used in the identification of biomarkers,^42^ we only measured one protein in this study, which may seem unconvincing. In future studies, we may confirm our results by simultaneously measuring serum levels of inflammatory factors. Additionally, only CHD patients without diabetes or kidney disease were recruited. Thus, the impact of other diseases and comorbidities on sTIMD4 remains a potential direction of further study. Finally, the causal relationship between serum sTIMD4 levels and CHD is not yet clear. Nevertheless, our study is novel as it measured sTIMD4 levels in patients with CHD for the first time and demonstrated that sTIMD4 is an independent risk factor for ACS. Furthermore, we provided evidence that the formation of sTIMD4 may be caused by the activation of p38 MAPK to promote the expression of ADAM17 and increase the cleavage of TIMD4 in the inflammatory environment. These findings provide a promising biomarker for CHD and shed light on the underlying mechanism of TIMD4 in atherosclerosis.

## 4 MATERIALS AND METHODS

### 4.1 Antibodies and reagents

Santa Cruz Biotechnology (Dallas, Texas, USA) provided the anti‐TIMD4 (Cat# SC‐390805), anti‐ADAM17 (Cat# SC‐390859), and TGF‐β1 (Cat# sc‐130348) antibodies. Affinity Biosciences (Cincinnati, Ohio, USA) provided the anti‐p‐NF‐κB (Cat# AF2006), anti‐IL‐6 (Cat# DF6087), anti‐IL‐10 (Cat# DF6894), and anti‐IL‐18 (Cat# DF6252) antibodies. ABclonal (Boston, MA, USA) provided anti‐NF‐κB (Cat# A16271), anti‐phosphorylated p38 MAPK (p‐p38 MAPK, Cat# AP0526), anti‐p38 MAPK (Cat# A14401), anti‐caspase‐1 (Cat# A0964), and anti‐IL‐1β (Cat# A16288) antibodies. Proteintech (Chicago, IL, USA) provided the anti‐TLR‐4 (Cat# 66350‐1‐Ig), anti‐TGF‐β1 (Cat# 21898‐1‐AP) antibodies, HRP‐conjugated GAPDH monoclonal antibody (Cat# HRP‐60004), and fluorescein‐conjugated AffiniPure Goat Anti‐Rabbit IgG (H+L) (Cat# SA00003‐2). MedChemExpress (Monmouth Junction, NJ, USA) provided TAPI‐1 (Cat# HY‐16657) and SB203580 (Cat# HY‐10256). Yiyuan Biotechnology (Guangzhou, China) provided ox‐LDL (Cat# YB‐002). Sigma–Aldrich (St. Louis, MO, USA) provided LPS (Cat# L2630).

### 4.2 Study population and design

We conducted a cross‐sectional study with human serum samples at the First Affiliated Hospital of University of Science and Technology of China starting from July 2022. The study was approved by the Ethics Committee of the First Affiliated Hospital of the University of Science and Technology of China (ethics acceptance number: 2022‐ky303) and was carried out in accordance with the ethical guidelines of the 1975 Declaration of Helsinki. Written informed consent was obtained from all participants.

Two groups were included in this study. In Group 1, patients with a high risk of CHD from the First Affiliated Hospital of the University of Science and Technology of China initially underwent coronary arteriography. performed by a team of experienced cardiologists. We excluded patients with diabetes or/and nephropathy, and then recruited 70 CHD patients, collecting blood samples from them. In addition, blood samples were also collected from 36 individuals (age ≥18 years) without any diseases, who were defined as healthy controls.

In Group 2, we recruited 81 patients (age ≥18 years) who were diagnosed with ACS and 44 patients (age ≥18 years) diagnosed with CCS, with exclusion of diabetes and/or nephropathy. The clinical syndromes of ACS include acute STEMI, acute NSTEMI, and UA. The diagnosis and classification of ACS are determined by clinical symptoms, electrocardiogram changes commensurate with acute myocardial ischemia and troponin elevation. Acute myocardial infarction (STEMI and NSTEMI) and UA are distinguished according to troponin elevation. STEMI is defined as 30 min of persistent chest pain at least, ST‐segment elevation of 0.1 mV in at least 2 consecutive leads, or a new left bundle branch block in 18‐lead electrocardiogram, as assessed by optical coherence tomography.^43^ According to the 2019 ESC Guidelines for the diagnosis and management of CCS, the CCS includes six common clinical cases of suspicious or confirmed CCS: (1) patients suspected CHD with stable angina symptoms or dyspnea; (2) patients with new heart failure or left ventricular insufficiency; (3) patients with stable symptoms within 1 year after ACS or with recent revascularization; (4) symptomatic or non‐symptomatic patients who have been newly diagnosed or had revascularization for more than 1 year; (5) patients with angina pectoris, suspected vasospasm or microvascular disease; (6) asymptomatic patients were detected with CHD by screening.^43^

### 4.3 Determination of sTIMD4 levels in human serum samples

The whole blood was collected in tubes without anticoagulant and centrifuged at ~600 *g* for 10 min at 4°C. The supernatant was collected as serum and stored in cryovials at −80°C. The level of serum sTIMD4 levels were measured using a human TIMD4 ELISA kit (Cat# CSB‐EL023546HU; Cusabio, Wuhan, China) following the manufacturer's instructions.

### 4.4 Cell culture

The RAW 264.7 mouse macrophage cell line was obtained from ATCC (Rockville, MD, USA), and cultured in RPMI 1640 medium supplemented with 10% fetal bovine serum and 1% penicillin (100 U/mL)–streptomycin (100 mg/mL), and in a 37°C humidified incubator with 95% air and 5% CO_2_. RAW 264.7 cells were treated with ox‐LDL (80 μg/mL), LPS (1 μg/mL), TAPI‐1 (1 μM), and SB203580 (10 μM) following previous studies and product instruction.^44^, ^45^

### 4.5 Quantitative real‐time PCR

Total RNA was extracted from cells using TRIzol reagent (Cat# 15596026; Invitrogen, Carlsbad, CA, USA). Purified RNA was reverse‐transcribed into cDNA using the RevertAid™ First Strand cDNA Synthesis kit (Cat# K1622; Thermo Fisher Scientific, Waltham, MA, USA), and the cDNA was analyzed by quantitative real‐time PCR (qRT‐PCR) using AceQ qPCR SYBR Green Master Mix (Cat# Q121‐02; Vazyme, Nanjing, China) and the corresponding primers (Table S1). The housekeeping gene used was β‐actin or GAPDH.

### 4.6 Immunoblotting and immunofluorescent staining

Cells were collected and lysed in RIPA buffer (Cybrdi, Gaithersburg, USA) supplemented with protease inhibitors (Roche Diagnostics, Indianapolis, IN, USA). The protein concentration was determined by a BCA protein assay kit (Cat# 23225; Thermo Fisher Scientific). The expression of TIMD4, ADAM17, p‐p38, p38, TLR‐4, p‐NF‐κB, p‐NF‐κB, IL‐6, IL‐10, IL‐18, TGF‐β1, caspase‐1, pro‐IL‐1β, IL‐1β protein in whole cellular extract, and sTIMD4 in cell culture medium were detected by Western blotting. The statistical analysis results of Western blotting band density are available in the Supplementary Information.

TIMD4 protein expression was also detected by immunofluorescent staining. RAW264.7 cells cultured on coverslips were fixed with 4% paraformaldehyde for 30 min. incubated with anti‐TIMD4 antibody overnight at 4°C and then incubated with Fluorescein‐conjugated goat anti‐rabbit IgG for 2 h at room temperature. The nuclei were then stained with DAPI solution, and the slices were sealed, followed by observation and photography with a fluorescence microscope (Leica, Wetzlar, Germany). Rabbit normal IgG was used to replace the anti‐TIMD4 antibody for negative control.

### 4.7 siRNA transfection

TIMD4 siRNA (Cat# siG1232692708), ADAM17 siRNA (Cat# siB1237142657‐1‐5), and NC siRNA (Cat# siN0000001‐1‐5) were synthetized by RIBO BIOTECHNOLOGY Co., Ltd (Guangzhou, China). The siRNA sequence for targeting mouse TIMD4 was 5′‐CCATGGACCTCGTGAAGAA‐3′ and the siRNA sequence for targeting mouse ADAM17 was 5′‐CTACAAGACCATAGAAAGT‐3′. RAW264.7 cells were transfected with 20 nM si‐TIMD4/si‐ADAM17 or scrambled control si‐NC using Lipofectamine™ RNAiMAX (Cat# 13778030; Thermo Fisher Scientific) in serum‐free medium when cell density reached approximately 50%. Following 24 h transfection, complete RPMI 1640 medium was added, and cells were cultured for an additional 24 h.

### 4.8 Statistical analysis

Continuous data were expressed as mean ± standard deviation or median (interquartile range), and normality was assessed using the Shapiro–Wilk test. Comparisons between two groups were made using Student's *t*‐test or nonparametric test as appropriate. Categorical variables were expressed as count (percent), and Pearson's chi‐squared test was used to compare the significance between the two groups. Logistic regression analysis was performed to determine the odds ratio and 95% CI of sTIMD4, with adjustments made for variables including age, sex, bodyweight, BMI, TG, T‐CHO, LDL‐C, HDL‐C, AI, smoking, fasting blood glucose, systolic and diastolic BP, hypertension, cerebrovascular disease, and hyperlipemia. The predictive ability of sTIMD4 for CHD was evaluated using area under the ROC curve, sensitivity, and specificity. Experimental data with more than two groups were analyzed using one‐way analysis of variance. A probability value of *p* < 0.05 was considered statistically significant. SPSS version 25 and GraphPad Prism 8 were used for all statistical analysis.

## AUTHOR CONTRIBUTIONS

Mengyao Wang, Yajun Duan, Jihong Han, and Likun Ma designed the study, drafted and edited the manuscript; Hui Zhang assisted with the ethics application for this study. Mengyao Wang and Ke Gong collected the data and performed most of the experiments. Mengyao Wang analyzed the data. Xinran Zhu, Shasha Chen, and Jie Zhou assisted with the experimental operation. All authors have read and approved the final manuscript.

## CONFLICT OF INTEREST STATEMENT

The authors declare no competing interests associated with this manuscript.

## ETHICS STATEMENT

This study was approved by the Ethics Committee of the First Affiliated Hospital of the University of Science and Technology of China (ethics acceptance number: 2022‐ky303). Written informed consent was obtained from all participants.

## Supporting information

Supporting Information

## Data Availability

All data are available from the corresponding authors upon request.
