# Supplementary material for: Identification of circulating T‐cell immunoglobulin and mucin domain 4 as a potential biomarker for coronary heart disease
Source: MedComm (2020). 2023 Jul 8;4(4):e320. doi: 10.1002/mco2.320 (PMC10329472; doi:10.1002/mco2.320)
Supplement: Supplementary file 1 — Supporting Information [file MCO2-4-e320-s001.docx]

**Identification of circulating** **T-cell immunoglobulin and mucin domain 4 as a potential biomarker for coronary heart disease**

Mengyao Wang^1,#^, Ke Gong^1,#^, Xinran Zhu^1^, Shasha Chen^1^, Jie Zhou^1^, Hui Zhang^2^, Jihong Han^1,3^, Likun Ma^2,^*, Yajun Duan^2,^*

^1^*Key Laboratory of Metabolism and Regulation for Major Diseases of Anhui Higher Education Institutes, Hefei University of Technology, Hefei, China;*

^2^*Department of Cardiology, The First Affiliated Hospital of USTC, Division of Life Sciences and Medicine, University of Science and Technology of China, Hefei, China;*

^3^*College of Life Sciences, State Key Laboratory of Medicinal Chemical Biology, Key Laboratory of Bioactive Materials of Ministry of Education, Nankai University, Tianjin, China;*

*Correspondence should be addressed to:

Likun Ma, MD

Department of Cardiology, The First Affiliated Hospital of the University of Science and Technology of China, Hefei, China

Tel: 86-18756967633; E-mail: lkma@ustc.edu.cn

Yajun Duan, PhD

Division of Life Sciences and Medicine, University of Science and Technology of China, Hefei, China

Tel: 86-13820168100; E-mail: yajunduan@ustc.edu.cn

**Table S1.** Sequences of primers derived from mouse for qRT-PCR

| **Genes** | **Forward** | **Backward** |
| --- | --- | --- |
| m-TIMD4 | 5’-CATGAAAGGGGCAGTCCTTA-3’ | 5’-CGTCTGCCTTCATCCTTCTC-3’ |
| m-ADAM17 | 5’-TTCACAAACACCTCCCCTTC-3’ | 5’-ACATCGAGAGTGGCCAGATT-3’ |
| m-IL-6 | 5’-TCCGAGGAGAACAAGCTGTC-3’ | 5’-CACTGGTCATGGCTGAGAAA-3’ |
| m-TLR-4 | 5’-GCCATTGGTATTGGGGCTTAC-3’ | 5’-CCCGACCAAGGACTTTGTTG-3’ |
| m-TNF-α | 5’-ACTGGGACGACATGGAAAAG-3’ | 5’-GTTCAGTGGTGCCTCTGTCA-3’ |
| m-TGF-β1 | 5’-GAGCCCCGGGGTGGAACAAGAT-3’ | 5’-AAAAGGTGGTGGGCAGGAGTAAGG-3’ |

**Table S2.** Logistic regression analyses of serum sTIMD4 levels for Group 1

| Model | Odds Ratio | 95% CI | *p*-value |
| --- | --- | --- | --- |
| Unadjusted | 1.007 | 1.003-1.010 | <0.001 |
| Model 1 | 1.007 | 1.002-1.011 | 0.007 |
| Model 2 | 1.008 | 1.001-1.014 | 0.024 |
| Model 3 | 1.008 | 1.001-1.015 | 0.021 |

Model 1 was adjusted for age, sex, weight, and BMI. Model 2 was adjusted for all factors in Model 1 plus LDL-C, HDL-C, T-CHO, TG and AI. Model 3 adjusted for all factors in Model 2 plus blood glucose, systolic and diastolic BP. 95% CI: 95% confidence intervals. *p*<0.05 was considered significant.

**Table S3.** Logistic regression analyses of serum sTIMD4 levels for Group 2

| Model | Odds Ratio | 95% CI | *p*-value |
| --- | --- | --- | --- |
| Unadjusted | 1.374 | 1.109-1.701 | 0.004 |
| Model 1 | 1.369 | 1.099-1.704 | 0.005 |
| Model 2 | 1.432 | 1.124-1.835 | 0.004 |
| Model 3 | 1.502 | 1.139-1.981 | 0.004 |

Model 1 was adjusted for age, sex, weight, and BMI. Model 2 was adjusted for all factors in Model 1 plus LDL-C, HDL-C, T-CHO, TG, AI and smoking. Model 3 adjusted for all factors in model 2, plus blood glucose, systolic and diastolic BP, hypertension, cerebrovascular disease, and hyperlipidemia. 95% CI: 95% confidence intervals. *p*<0.05 was considered significant.

**Table S4.** Spearman correlations for clinical characteristics and serum sTIMD4 values

*CHD patients and healthy subjects*

| Clinical characteristics | Spearman's rho | *p*-value |
| --- | --- | --- |
| Age | **0.221** | **0.023** |
| Bodyweight | **0.391** | **<0.001** |
| BMI | **0.356** | **<0.001** |
| AI | **0.356** | **<0.001** |
| Systolic BP | 0.146 | 0.136 |
| Diastolic BP | 0.141 | 0.149 |
| Clinical blood measurements |  |  |
| Blood glucose | -0.083 | 0.397 |
| LDL-C | -0.011 | 0.908 |
| HDL-C | **-0.394** | **<0.001** |
| T-CHO | -0.044 | 0.652 |
| TG | **0.372** | **<0.001** |

*Patients with CCS and ACS*

| Clinical characteristics | Spearman's rho | *p*-value |
| --- | --- | --- |
| Age | 0.058 | 0.523 |
| Bodyweight | -0.101 | 0.264 |
| BMI | -0.105 | 0.245 |
| AI | 0.134 | 0.136 |
| Systolic BP | 0.058 | 0.522 |
| Diastolic BP | 0.015 | 0.867 |
| Clinical blood measurements |  |  |
| Blood glucose | -0.029 | 0.75 |
| LDL-C | 0.076 | 0.403 |
| HDL-C | -0.106 | 0.24 |
| T-CHO | 0.051 | 0.575 |
| TG | -0.012 | 0.896 |

BMI, body mass index; LDL-C, low-density lipoprotein cholesterol; HDL-C, high-density lipoprotein cholesterol; T-CHO, total cholesterol; TG, total triglyceride; AI, atherosclerosis index. *p*<0.05 was considered significant.

**Table S5.** Diagnostic performance of sTIMD4 in distinguishing CHD

| Cut-off Value, ng/ml | Sensitivity, % | Specificity, % | Accuracy, % | PPV, % | NPV, % |
| --- | --- | --- | --- | --- | --- |
| 0.34 | 74.9 | 66.7 | 74 | 93 | 33.8 |

NPV, negative predictive value; PPV, positive predicting value; Optimal threshold value obtained from the data, which was the threshold leading to the maximum summation of sensitivity and specificity.

**
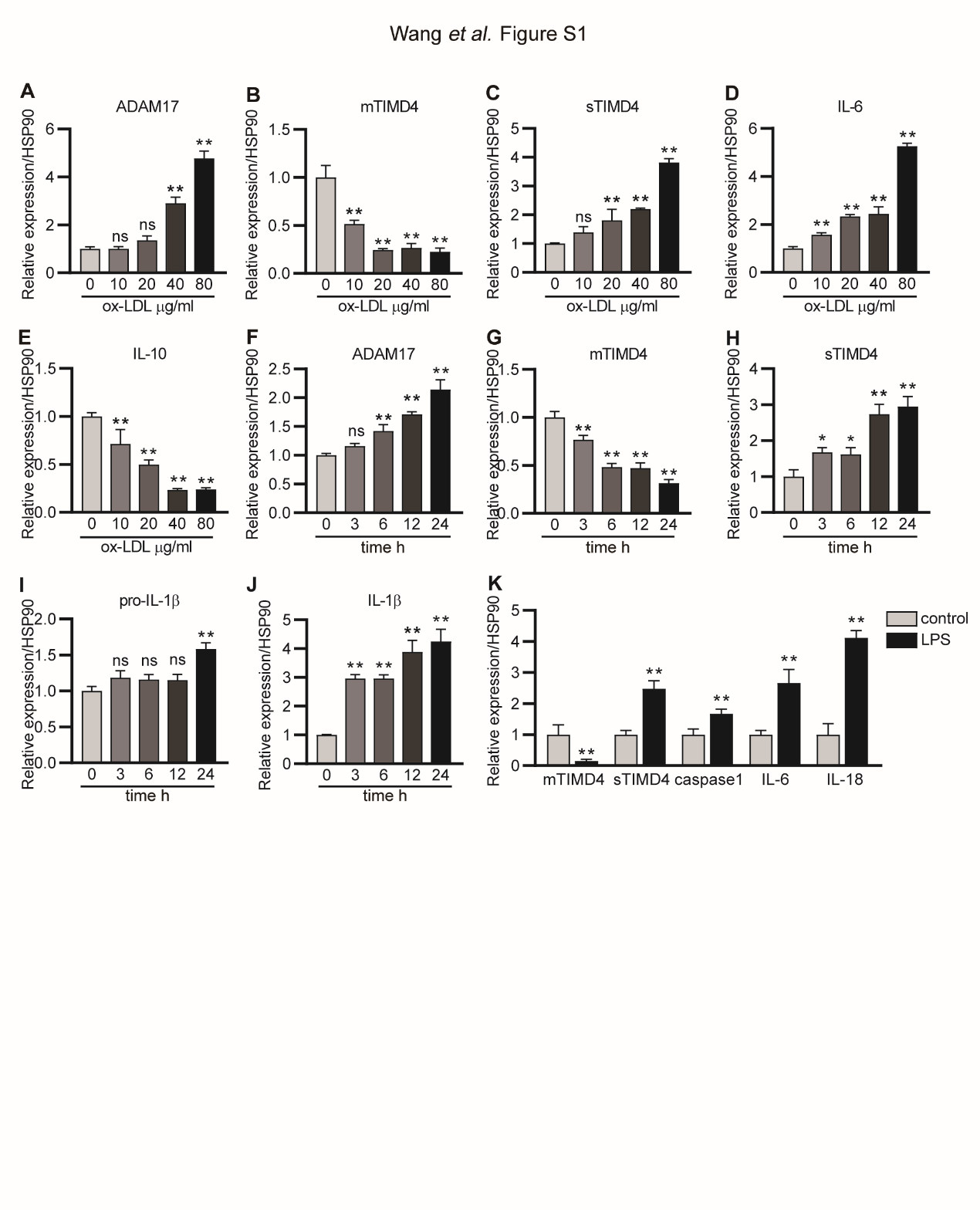
**

**Figure S1.** The statistical results of quantitative analysis of protein band density for Figure 3A-C.

All the Western blotting images for Figure 3A-C were conducted analysis of band density, and normalized to the density of HSP90 in the corresponding samples. (A-E) ADAM17, mTIMD4, sTIMD4, IL-6 and IL-10 in Figure 3A; (F-J) ADAM17, mTIMD4, sTIMD4, pro-IL-1β and IL-1β in Figure 3B; (K) mTIMD4, sTIMD4, caspase-1, IL-6 and IL-18 in Figure 3C. ns: not significant, **p*<0.05, ***p*<0.01, n=3.

**
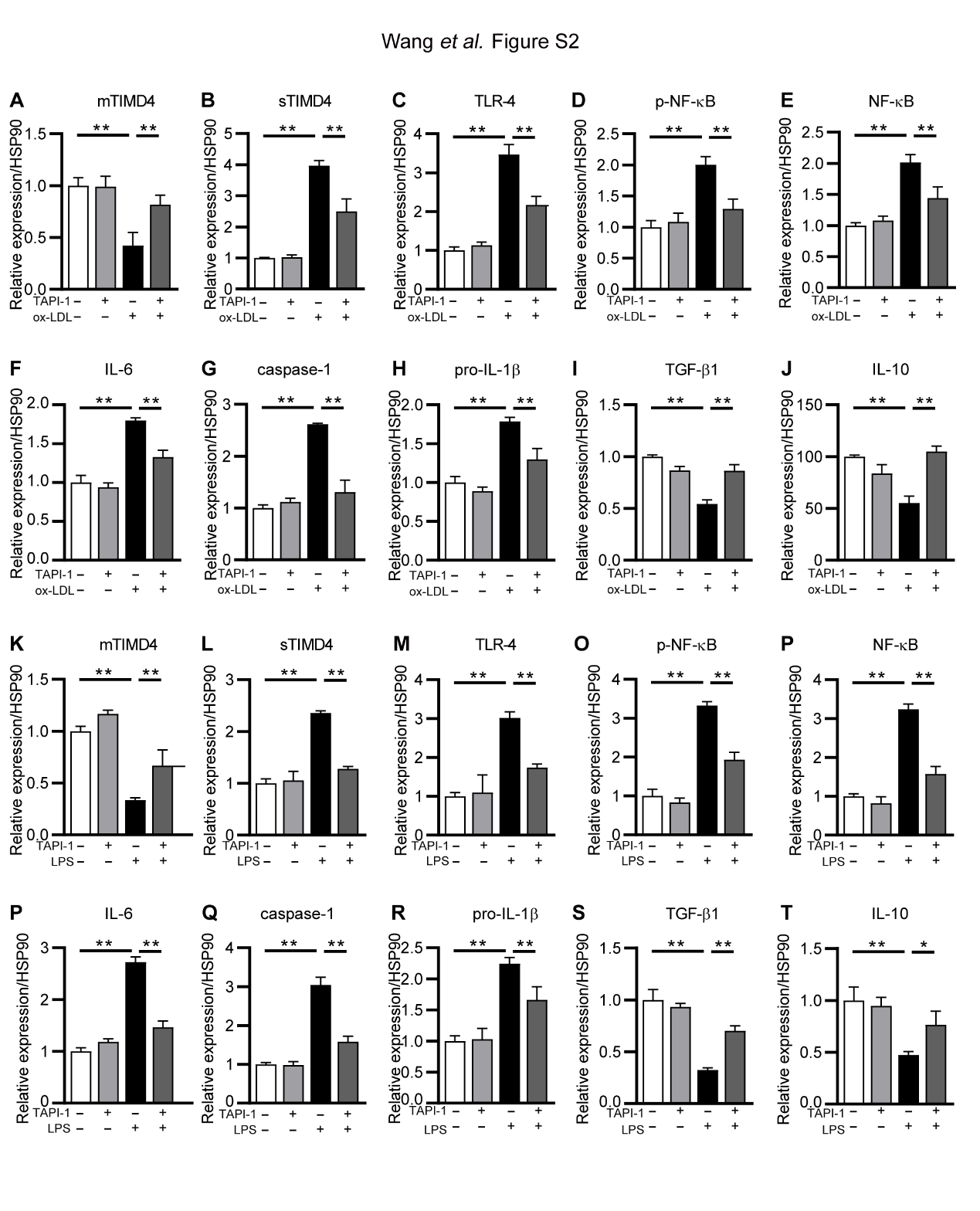
**

**Figure S2.** The statistical results of quantitative analysis of protein band density for Figure 4C-F and 4K-N.

All the Western blotting images for Figure 4C-F and 4K-N were conducted analysis of band density, and normalized to the density of HSP90 in the corresponding samples. (A-J) mTIMD4, sTIMD4, TLR-4, p-NF-κB, NF-κB, IL-6, caspase-1, pro-IL-1β, TGF-β1 and IL-10 in Figure 4C-F; (K-T) mTIMD4, sTIMD4, TLR-4, p-NF-κB, NF-κB, IL-6, caspase-1, pro-IL-1β, TGF-β1 and IL-10 in Figure 4K-N. **p*<0.05, ***p*<0.01, n=3.


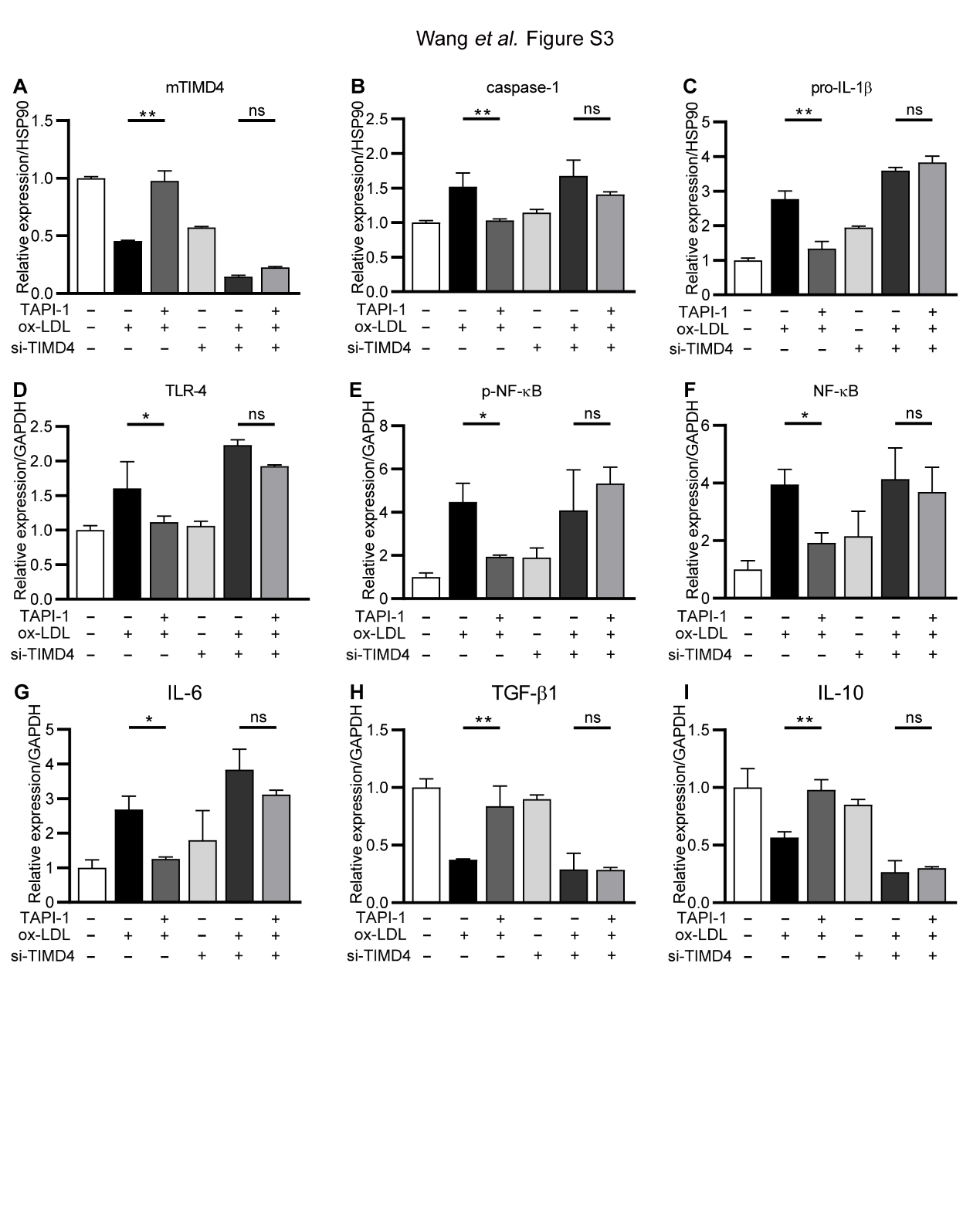


**Figure S3.** The statistical results of quantitative analysis of protein band density for Figure 4O-P.

All the Western blotting images for Figure 4O-P were conducted analysis of band density, and normalized to the density of HSP90 or GAPDH in the corresponding samples. (A-I) mTIMD4, caspase-1, pro-IL-1β, TLR-4, p-NF-κB, NF-κB, IL-6, TGF-β1 and IL-10 in Figure 4O-P. ns: not significant, **p*<0.05, ***p*<0.01, n=3.

**
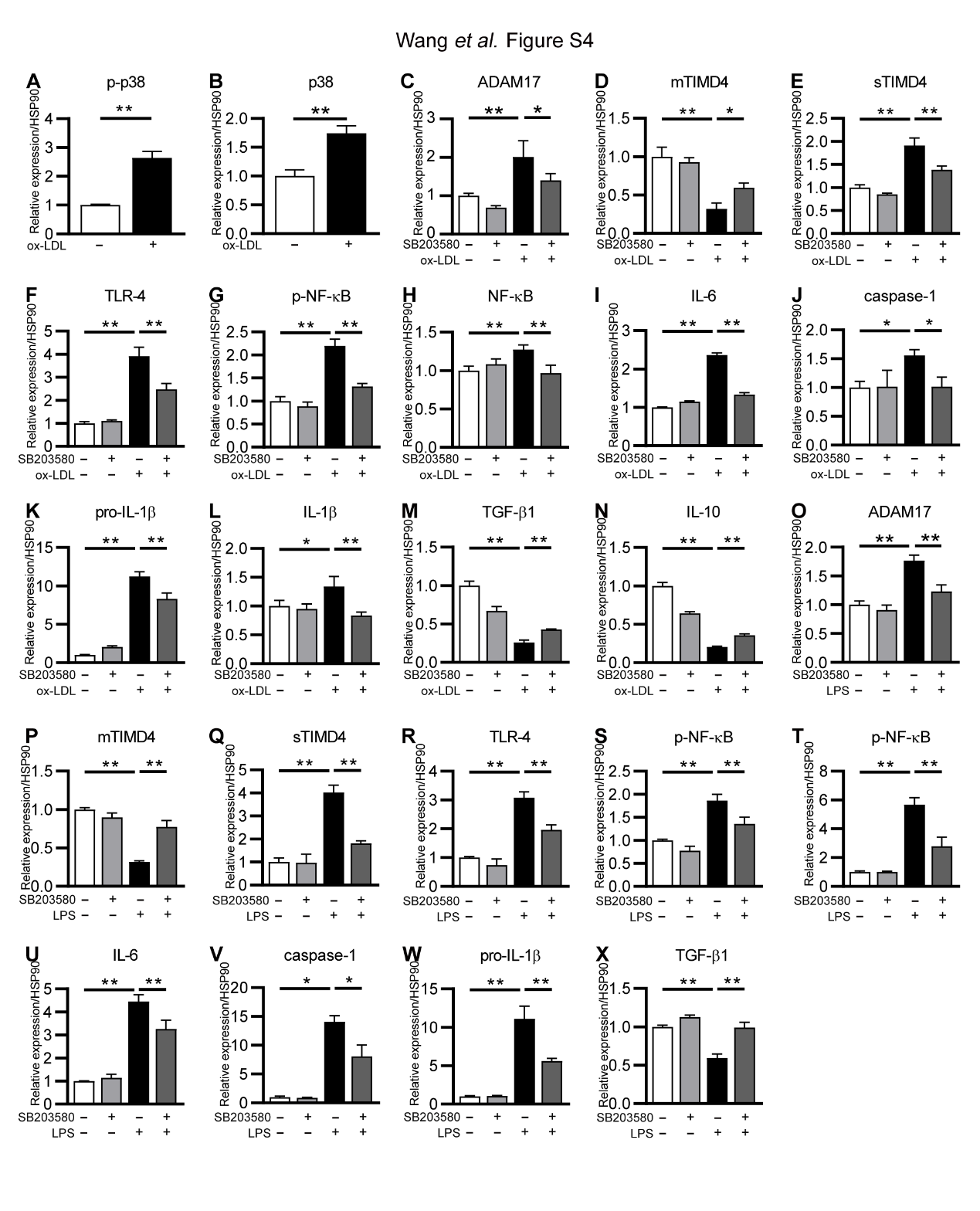
**

**Figure S4.** The statistical results of quantitative analysis of protein band density for Figure 5A-I.

All the Western blotting images for Figure 5A-I were conducted analysis of band density, and normalized to the density of HSP90 in the corresponding samples. (A-N) p-p38, p38, ADAM17, mTIMD4, sTIMD4, TLR-4, p-NF-κB, NF-κB, IL-6, caspase-1, pro-IL-1β, IL-1β, TGF-β1 and IL-10 in Figure 5A-E. (O-X) mTIMD4, sTIMD4 in medium, ADAM17, TLR-4, NF-κB, p-NF-κB, IL-6, caspase-1, pro-IL-1β and TGF-β1 in Figure 5F-I. **p*<0.05, ***p*<0.01, n=3.


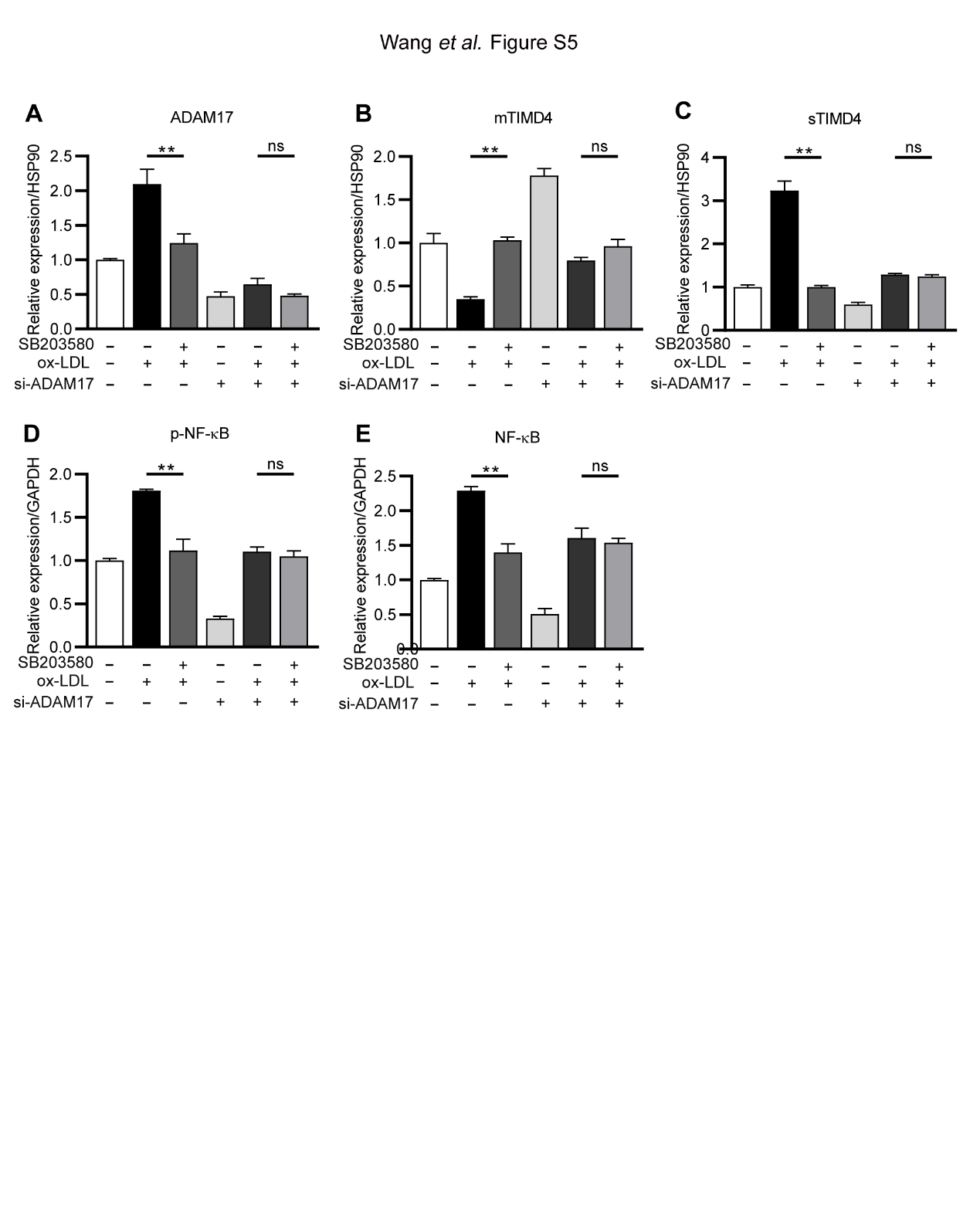


**Figure S5.** The statistical results of quantitative analysis of protein band density for Figure 5J-K.

All the Western blotting images for Figure 5J-K were conducted analysis of band density, and normalized to the density of HSP90 or GAPDH in the corresponding samples. (A-E) ADAM17, mTIMD4, sTIMD4, p-NF-κB, NF-κB in Figure 5J-K. ns: not significant, **p<0.01, n=3.
